# Deciphering the Prognostic Landscape of Esophageal Adenocarcinoma: A PANoptosis-Related Gene Signature

**DOI:** 10.7150/jca.102180

**Published:** 2025-01-01

**Authors:** Haijing Fu, Mengyan Liu, Huiyu Li, Li Yu, Haizhu Song, Xiaoyuan Chu, Wei Bao

**Affiliations:** 1 Department of Medical Oncology, Jinling Hospital, Nanjing Medical University, Nanjing, 210000, China.; 2 Department of Medical Oncology, Nanjing Jinling Hospital, Affiliated Hospital of Medical School, Nanjing University, Nanjing, 210000, China.; 3 Department of Medical Oncology, Jinling Hospital, Nanjing University of Chinese Medicine, Nanjing, 210000, China.; 4 Department of Medical Oncology, Jinling Hospital, The First School of Clinical Medicine, Southern Medical University, Nanjing, 210000, China.; 5 Department of Pathology, Nanjing Jinling Hospital, Affiliated Hospital of Medical School, Nanjing University, Nanjing, 210000, China.; 6 Department of Pathology, Jinling Hospital, The First School of Clinical Medicine, Southern Medical University, Nanjing, 210000, China.

**Keywords:** Esophageal Adenocarcinoma (EAC), PANoptosis, Risk Scoring Model, Immune Infiltration, tumor microenvironment, MMP12.

## Abstract

**Backgrounds:** Esophageal adenocarcinoma (EAC) remains a challenging malignancy with low survival rates despite advances in treatment. Understanding the molecular mechanisms and identifying reliable prognostic markers are crucial for improving clinical outcomes.

**Methods:** We conducted a comprehensive bioinformatics analysis utilizing TCGA, GTEx, and GEO datasets to identify PANoptosis-related genes (PRGs) associated with EAC. From this analysis, we developed a prognostic risk score model based on 8 prognostically significant differentially expressed PRGs. This model was externally validated and compared with traditional staging methods. Functional analyses, including gene expression profiling, pathway enrichment analysis, and immune infiltration assessment, were conducted to elucidate the biological mechanisms influencing prognosis. To identify PANoptosis-related hub genes, we employed Weighted Gene Co-expression Network Analysis (WGCNA). The expression profiles of the hub gene were examined using reverse transcription-quantitative PCR (RT-qPCR) and western blotting. Furthermore, the effects of the hub genes knockdown or overexpression on EAC cell behavior were verified through in vitro experiments, including cell counting kit (CCK)-8, transwell and wound healing assay.

**Results:** The prognostic risk score model effectively predicts patient outcomes, supported by principal component analysis (PCA) and receiver operating characteristic (ROC) curves. The resulting prognostic nomogram, which integrates clinical features and the risk score, outperforms traditional staging systems, offering enhanced predictive accuracy. WGCNA identified gene modules significantly correlated with EAC clinical traits, highlighting the biological relevance of these genes to disease progression. Functional enrichment analyses shed light on significant biological processes and pathways associated with high-risk EAC, including lipid metabolism and hormone transport. Immune infiltration analysis revealed distinct immune profiles between risk groups, pinpointing potential immunotherapeutic targets. Furthermore, drug sensitivity analysis indicated specific compounds that may be more effective in high-risk groups. Notably, MMP12 emerged as a key mediator and further experimental results revealed that the lower the degree of cell differentiation, the higher the expression level of MMP12 in EAC. The knockdown of MMP12 significantly inhibited cell proliferation and migration.

**Conclusions:** Our findings present a validated risk scoring model and prognostic nomogram as valuable tools for predicting patient outcomes and guiding personalized treatments in EAC. This study underscores the potential of molecular clustering and PANoptosis-based prognostic features in predicting patient survival and understanding the tumor microenvironment's complexity, especially the metabolic and immune profiles, in EAC. These insights enhance our understanding of PANoptosis in EAC and provide new avenues for its diagnosis and therapy.

## Introduction

Esophageal cancer is among the most common malignant tumors of the gastrointestinal tract, with its incidence rates rapidly increasing worldwide. In 2020, the global incidence of esophageal cancer ranked seventh among all malignancies, with its total mortality ranking sixth^1^. Histologically, esophageal cancer predominantly presents as squamous cell carcinoma in Asia, accounting for over 90% of cases, whereas adenocarcinoma predominates in the United States and Europe, comprising about 80%^2^. The development of EAC is closely associated with alcohol consumption, smoking, obesity, and gastroesophageal reflux, yet its precise carcinogenic mechanisms remain unclear^3^. Despite significant advancements in treatments, including esophagectomy, chemotherapy, molecular targeted therapy, and immune checkpoint blockade therapy, the 5-year survival rate for EAC remains below 20%^4^. Hence, investigating key genes in EAC and establishing an accurate prognostic model are crucial for early screening and precise treatment.

Cell death is a fundamental biological process essential for maintaining homeostasis and plays a critical role in both inflammatory responses and oncogenesis^5^. Programmed cell death (PCD), encompassing pyroptosis, apoptosis, necroptosis, and ferroptosis, is a regulated and active form of cellular death^6^. PANoptosis, a recently discovered PCD pathway, results from the interplay and concurrent activation of apoptosis, pyroptosis, and necroptosis^7^. This process is mediated by the PANoptosome, a cytoplasmic multiprotein complex containing Receptor-Interacting Protein Kinase 1 (RIPK1), Apoptosis-Associated Speck-Like Protein Containing a CARD (ASC), and caspase-8 (CASP8)^8^. CASP8 acts as a molecular switch, coordinating these three modes of cell death and triggering pro-inflammatory cell death^9^. Dysregulated cell death and inflammation are linked to tumorigenesis, PANoptosis plays a crucial role in tumor initiation and progression^10^. For instance, the PANoptosis-related lncRNA SNHG7 is involved in chemoresistance and metastasis in colon adenocarcinoma^11^, and it also contributes to antitumor defenses^12^, ^13^. Given its dual role in tumor biology, PANoptosis has become a focus of recent research^14^, ^15^, with studies identifying potential biomarkers for various cancers^16^, ^17^.

Although some studies have identified the roles of pyroptosis and necroptosis in EAC^18^, ^19^, the relationship between PANoptosis and EAC, especially the role of PRGs in tumor prognosis and microenvironment, remains unclear. Therefore, we performed a comprehensive evaluation of PRGs in EAC and created a detailed prognostic model. This model includes 8 key genes and integrates clinical pathological features. Through the model, we identified differences in prognosis, immune microenvironment, and drug sensitivity among different risk groups. Finally, through WGCNA identified PANoptosis-related hub gene and verified its expression levels, as well as influence on tumor invasion and metastasis.

## Methods

### Data acquisition and pre-processing

Eighty-eight EAC samples were selected from 182 ESCA cases in the TCGA database (https://portal.gdc.cancer.gov/repository). Given the scarcity of non-tumor samples in TCGA-ESCA, RNA-Seq data and clinical information for 652 normal esophageal samples were obtained from the GTEx project via the UCSC Xena platform (https://xena.ucsc.edu/). Data normalization and processing were performed using R version 4.2.3. For model validation, we used the GEO dataset GSE13898, which includes comprehensive clinicopathological data, as an independent cohort. Batch effects were adjusted using the limma R package^20^.

### Acquisition of Differentially Expressed PRGs (DEPRGs) and identification of prognostic DEPRGs

We compiled a list of 624 prognostic regulatory genes (PRGs) from existing research^21^-^24^, detailed in Supplementary Table S1. Redundant genes were removed to consolidate various gene sets. Differentially expressed genes (DEGs) between tumor and normal tissues were identified using the limma R package with thresholds of adjusted p-value < 0.05 and |log2 fold change| ≥ 1. Univariate Cox regression analysis selected PRGs with potential prognostic value (p-value < 0.05). Mutation frequencies of these DEPRGs were analyzed using the cBioPortal for Cancer Genomics. Gene interactions were mapped using GeneMANIA database (https://genemania.org/), and a chromosome map was created with the “RCircos” package. Visualizations including volcano plots, heatmaps, and Venn diagrams were generated using ggplot2, complex Heatmap, and Venn R packages, respectively^25^.

### Constructing and validating PANoptosis-related prognostic model

Patients from TCGA were used as the training cohort, while those from the GEO served as the validation cohort. To prevent overfitting of the prognostic signature, the glmnet package was employed to perform least absolute shrinkage and selection operator (LASSO) regression, resulting in the identification of eight prognostic-related DEPRGs. The estimated regression coefficients were weighted and combined with the expression values of prognostic-related DEPRGs to formulate the risk score for the prognostic gene signature as follows:



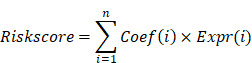



The risk score for each EAC patient was calculated using this formula, and patients were stratified into high- and low-risk groups based on the median risk score. PCA was conducted to examine the distribution across these groups. Furthermore, we investigated the correlation between the PANoptosis-related risk score and overall survival of EAC patients using the survival package for survival analysis. The prognostic validity of the PANoptosis-related risk scores was further assessed by constructing time-dependent ROC curves with the Survival ROC package. The prognostic value of our model was also verified in the validation set GSE13898.

### Development of nomogram

Prognostic nomograms were developed to predict overall survival (OS) in patients with EAC, utilizing gender, age, tumor stage and risk score. These nomograms were constructed using the rms package in R. The accuracy of the nomograms was assessed by comparing predicted versus actual 1-, 2-, and 3-year survival probabilities through the use of calibration diagrams. This approach provided a visual assessment of the model's predictive performance.

### Screening for prognosis-related key PRGs

To find key prognostic genes linked to PANoptosis in esophageal adenocarcinoma (EAC), we used WGCNA on TCGA data from high- and low-risk groups. We constructed a weighted gene network^26^, performed hierarchical clustering to define gene modules, and correlated these modules with risk scores. One module showed a strong positive correlation with the risk score and was selected for further analysis. Key genes were identified by intersecting this module with previously known PANoptosis-related genes, and a Venn diagram was used to highlight genes related to both traits and expression.

### Gene Oncology (GO) and Kyoto Encyclopedia of Genes and Genomes (KEGG) analyses

To understand the functions and pathways related to the DEPRGs, we conducted GO and KEGG analyses. GO categorizes gene functions into molecular function (MF), biological process (BP), and cellular component (CC). KEGG provides insights into high-level biological functions^27^. We used reference gene sets including C2KEGG, Hallmark, and C5GO, and visualized the results with the ggplot2 package and the Bioconductor platform. Statistical significance was determined with p-values and q-values below 0.05.

### Functional enrichment analysis

We used R packages “cluster Profiler” and “ggplot2” for Over-Representation Analysis (ORA) and Gene Set Enrichment Analysis (GSEA), drawing from GO, KEGG, and REACTOME databases. The analysis compared high- and low-risk score groups, with an absolute log2FC ≥ 1 and FDR < 0.05. The p-values were adjusted using the Benjamini-Hochberg (BH) method. The top five significantly enriched pathways were selected for further study. GSVA and ssGSEA were conducted using the “GSVA” and “GSEABase” packages in R^28^, ^29^ to assess the biological behavior of patients in different risk score groups based on gene sets from MSigDB(https://www.gsea-msigdb.org/gsea/msigdb/).

### Immune landscape analysis

Immune component profiles were assessed using several algorithms, including TIMER^30^, QuanTIseq^31^, CIBERSORT^32^, xCell^33^, MCPcounter^34^, and EPIC^35^. Visualization of these profiles was facilitated by the IOBR R packages^36^. ssGSEA was employed to calculate the scores for immune function and infiltration of immune cell subsets. Boxplots were utilized to display the differential expression of common immune checkpoints among subgroups. These checkpoints included the T-cell immune receptor with immunoglobulin and ITIM domains (TIGIT), programmed death 1 (PD-1), programmed death-ligand 1 (PD-L1), cytotoxic T lymphocyte-associated protein 4 (CTLA4), and members of the tumor necrosis factor (TNF) receptor superfamily. A p-value of less than 0.05 was considered statistically significant for indicating differences in the aforementioned indicators between the different groups.

### Analysis of drug susceptibility and prediction of small-molecule compounds

To investigate the association between two risk groups and drug sensitivity, the oncoPredict package was employed to predict the half-maximal drug inhibitory concentration (IC50) in EAC patients. These approaches initially built statistical models from gene expression and drug sensitivity data in cancer cell lines from Genomics of Drug Sensitivity in Cancer (GDSC) databases, and then applied these models to gene expression data of high- and low-risk groups.

### Cell culture and MMP12 knockdown and overexpression

The human normal esophageal cell line HET-1A (iCell-h333, iCell Bioscience Inc, Shanghai, China) and the esophageal adenocarcinoma (EAC) cell lines OE19 (iCell-h391, iCell Bioscience) and SKGT-4 (iCell-h482, iCell Bioscience) were each cultured in their respective specialized media (iCell-h333-001b, iCell-h391-001b, iCell-h482-001b) within an incubator maintained at 37°C with a 5% CO2 atmosphere. MMP12 small interfering RNA (siRNA) (104022, ThermoFisher, Waltham, MA, USA), Negative Control siRNA (AM4611; ThermoFisher) and overexpression plasma (GENECHEM, Shanghai, China) were transfected into OE19 and SKGT-4 cells using Lipofectamine 3000(Thermo Fisher). After 48 h transfection, the cells were harvested for further studies.

### Quantitative real-time PCR experiment

Total RNA was extracted from cells using TRIzol reagent (ThermoFisher). Subsequently, 500ng of total RNA was reverse-transcribed into cDNA in a 10 μL reaction volume using the TRUEscriptRT kit (Proteinbio, Nanjing, China). Specific primers were commercially synthesized (Proteinbio), and quantitative PCR (qPCR) was conducted using Universal SYBR Green qPCR Supermix (US EVERBRIGHT, Suzhou, China) on a 7500 Real-time PCR System (Applied Biosystems). Relative mRNA expression levels were normalized to GAPDH and quantified using the 2-ΔΔCT method. Each qPCR reaction was performed in triplicate. The sequences of the PCR primers (OriGene Wuxi Biotechnology Co., Ltd, Wuxi, China): Forward Sequence GATGCTGTCACTACCGTGGGAA; Reverse Sequence CAATGCCAGATGGCAAGGTTGG.

### Western blotting

Total proteins were extracted from SKGT-4 and OE19 cells using ice-cold radioimmunoprecipitation assay (RIPA) cell lysis buffer, supplemented with protease inhibitors (Sigma). Equal amounts of protein lysates were separated using 12% sodium dodecyl sulfate-polyacrylamide gel electrophoresis (SDS-PAGE). Then, the concentration was determined using the BCA protein assay kit (Beyotime Biotechnology Institute). The protein samples were transferred to PVDF membranes soaked in methanol and sealed with 5% bovine serum albumin at room temperature for 30 min. The membranes were mixed with primary antibodies against MMP12 (cat. no. ab52897; 1:1000 dilution; Abcam) and GAPDH (cat. no. ab181602; 1:10,000 dilution; Abcam) at 4°C overnight. The next day, the secondary antibodies conjugated to horseradish peroxidase (1:5000; Santa Cruz Biotechnology, Inc.) were added into the membranes for another 2 h incubation at room temperature. Lastly, we employed the enhanced chemiluminescence (ECL) kit to detect the protein signal.

### Cell counting assay

Cells in logarithmic growth phase were prepared as a cell suspension at a density of 5×10^3^ cells/mL. A 100 μL cell suspension was inoculated and incubated in each well of a 96-well plate. At 1 to 9 days after inoculation, 10 μL of cell counting kit-8 solution was added to each well and incubated for 1-2 h. The absorbance at 490 nm was measured with a microplate reader.

### Scratch wound healing and Transwell cell invasion assay

Cells in logarithmic growth phase were inoculated in a six-well plate. When cell density reached 90%, a straight line was drawn in the center of the well using a 200-μL pipette tip. Cells and debris were removed by washing with phosphate-buffered saline. Serum-free medium was added to each well, and the plate was incubated. Photographs were taken at 0 and 12 h. To evaluate the invasive capabilities of cells, Transwell assays were conducted. The upper chamber was coated with Matrigel (BD Bioscience, San Diego, CA, USA) at a 1:8 dilution. Subsequently, 30,000 cells in serum-free medium were seeded into the upper chamber, while the lower chamber received 750 µL of medium containing 10% FBS to promote cell migration across the membrane. After a 36-hour incubation, cells were fixed with 4% paraformaldehyde (PFA) for 30 minutes. Non-invading cells on the upper side of the membrane were removed using cotton swabs, and the remaining cells were stained with 0.1% crystal violet for 20 minutes. Cells were counted in five randomly selected fields at 100× magnification. All procedures were replicated three times.

### Statistical analysis

Data analysis and visualization were conducted using R (version 4.2.3). Initially, differences between two groups with normally distributed data were assessed using the t-test. For data in two groups not conforming to normal distribution, the Wilcoxon rank-sum test was applied. Subsequently, correlations were determined using either Spearman or Pearson analysis, depending on the distribution characteristics of the data. Survival durations for patients in different groups were compared using KM survival analysis. A p-value of less than 0.05 was considered to indicate statistical significance.

## Results

### Identification of 18 prognostic-related DEPRGs in EAC

**Figure 2:**
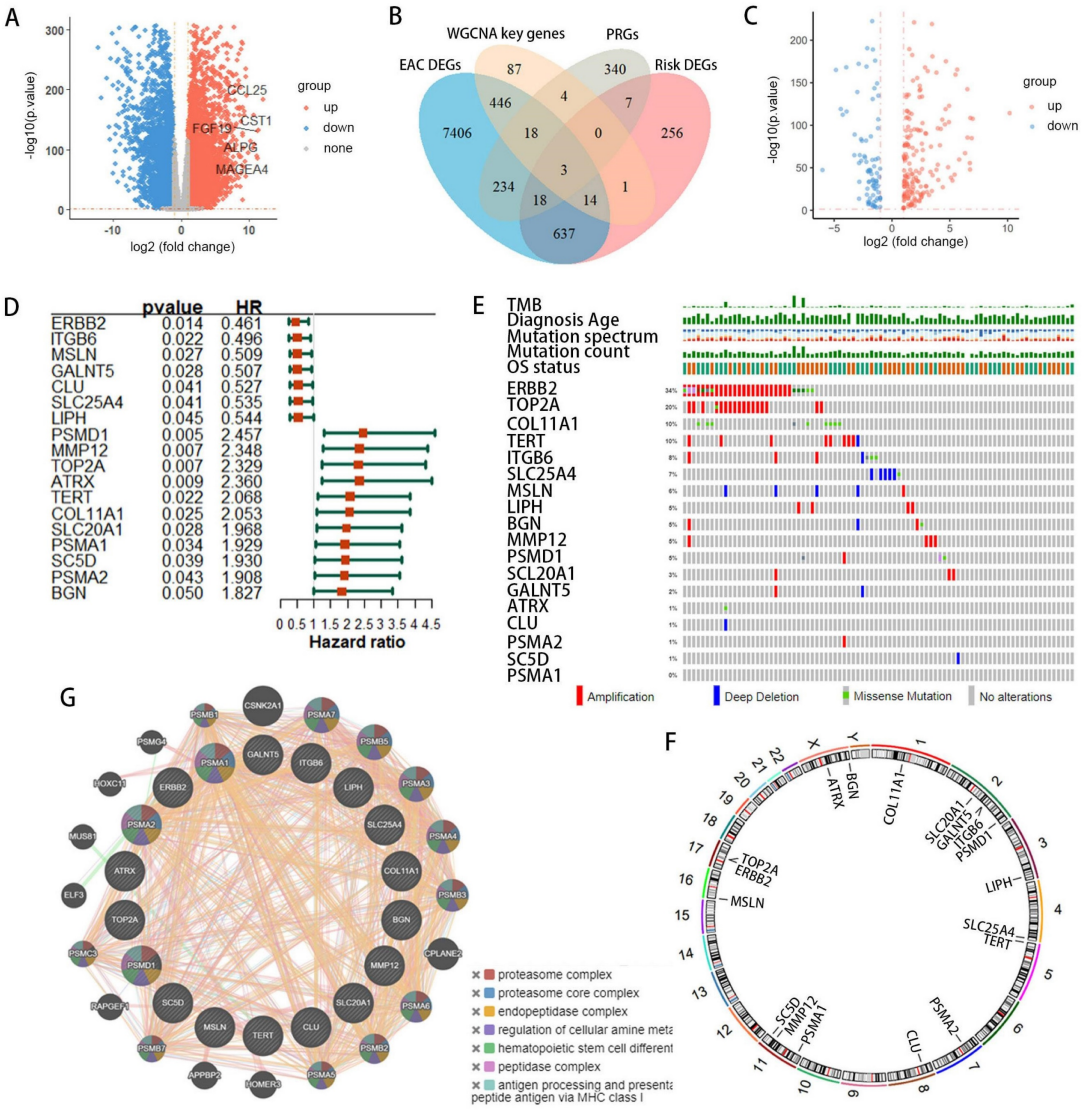
Identification of prognostic-related DEPRGs in EAC patients using TCGA and GTEx databases. (**A**) Volcano plot illustrating DEGs between normal and tumor samples. (**B**) Venn diagram delineating the core prognostic-related DEPRGs among EAC-DEGs, PRGs, WGCNA key genes, and risk-related DEGs. (**C**)Volcano plot depicting DEPRGs. (**D**) Univariate Cox regression analysis forest plot correlating 18 prognostic-related DEPRGs with overall survival (OS). (**E**) Mutation frequencies of the 18 PRGs within the TCGA-EAC cohort. (**F**) Location of the18 prognostic-related DEPRGs on the chromosome. (**G**) Co-expression network of 18 prognostic-related DEPRGs was shown by GeneMANIA.

We incorporated 88 EAC samples from the TCGA database and 652 normal esophageal samples from the GTEx database, totaling 720 samples as a training set. After batch correction and comparison of tumor versus normal esophageal samples, 8,776 genes showed significant differential expression (4,685 DEGs upregulated and 4,091 DEGs downregulated) (Figure 2A). By reviewing PRGs studies in the cancer field over the past three years, and after intersection and deduplication, we identified 624 PRGs (Table S1). Intersecting these PRGs with the 8,776 DEGs in EAC (Figure 2B), we obtained 273 DEPRGs, with 174 genes upregulated and 99 downregulated (Figure 2C). Univariate Cox regression was performed on the 273 DEPRGs, identifying 18 genes significantly associated with prognosis, including ATRX, BGN, CLU, COL11A1, ERBB2, GALNT5, ITGB6, LIPH, MMP12, MSLN, PSMA1, PSMA2, PSMD1, SC5D, SLC20A1, SLC25A4, TERT, and TOP2A (Figure 2D). To understand the mutation spectrum of these prognostic-related DEPRGs, we examined their mutation rates through the cBioPortal database (Figure 2E), where gene amplification, deep deletion, and missense mutations were the most common mutation types. In addition, we identified the locations of 18 differential genes on the 23 chromosomes (Figure 2F). Furthermore, we investigated the interactions between these PRGs and transcription proteins through a protein-protein interaction (PPI) network (Figure 2G). A total of 20 genes participated in this network, including MMP12, several of which were identified as key genes.

### Construction and evaluation of a risk score model

**Figure 3:**
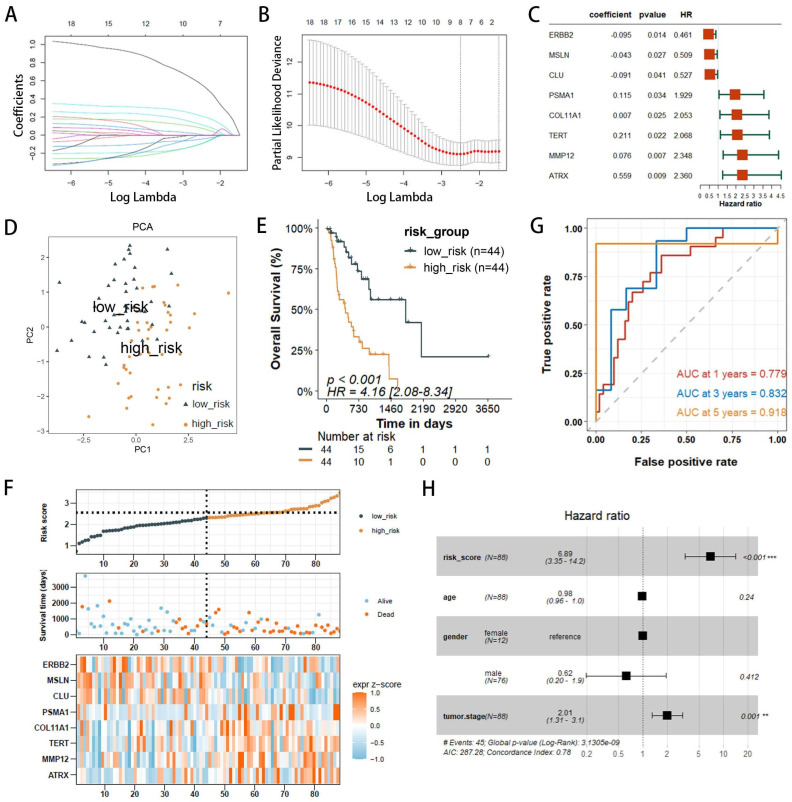
Development of PANoptosis-related prognostic models for EAC patients within the TCGA/GTEx cohorts. (**A, B**) LASSO coefficient profiles and penalty plots for the model incorporating 18 prognostic genes. (**C**)Univariate Cox regression analysis forest plots correlating 8 prognostic-related DEPRGs with overall survival (OS). (**D**) Principal component analysis (PCA) differentiating high- and low-risk groups in the TCGA cohort. (**E**) Kaplan-Meier (KM) survival curves comparing OS among patients in the two risk groups. (**F**) Distributions of risk (high or low), survival status of EAC patients, and expression levels of eight risk genes associated with PANoptosis. (**G**) Receiver operating characteristic (ROC) curves evaluating the predictive accuracy of the risk score within the TCGA cohort. (**H**) Outcomes of multivariate Cox regression analysis of OS to assess the prognostic independence of the risk score.

The 18 prognostic-related DEPRGs were further subjected to Lasso analysis (Lasso Coefficients (Figure 3A), Lasso Deviance (Figure 3B)), yielding 8 PRGs with prognostic relevance (ATRX, TERT, PSMA1, ERBB2, CLU, MMP12, MSLN, and COL11A1) (Figure 3C) that were used to construct a risk score model (Table S2).

The risk score model is as follows: Risk Score = 0.559×Expr_ATRX_+0.211×Expr_TERT_+0.115×Expr_PSMA1_-0.095×Expr_ERBB2_-0.091×Expr_CLU_+0.076×Expr_MMP12_-0.043×Expr_MSLN_+0.0065×Expr_COL11A1_.

Patients with EAC were divided into high-risk (n=44) and low-risk (n=44) groups based on the median risk score. PCA indicated that the model had good discriminative ability (Figure 3D). Further analysis of the survival rates for the high- and low-risk groups revealed a negative correlation between the risk score and patient overall survival rates (Figure 3E). Additionally, the survival status and risk plot (Figure 3E, F) demonstrated the same results. The prognostic model's diagnostic efficacy was evaluated using ROC curves. The AUC values for 1-, 3-, and 5-year survival rates of the model were 0.779, 0.832, and 0.918, respectively (Figure 3G). Moreover, in multivariate Cox regression analysis, the hazard ratio (HR) for the risk score was 6.89 (95%CI=3.35~14.2), significantly outperforming traditional risk factors such as tumor staging (HR=2.01) (Figure 3H).

### Validation of the model and construction of prognostic nomograms

**Figure 4:**
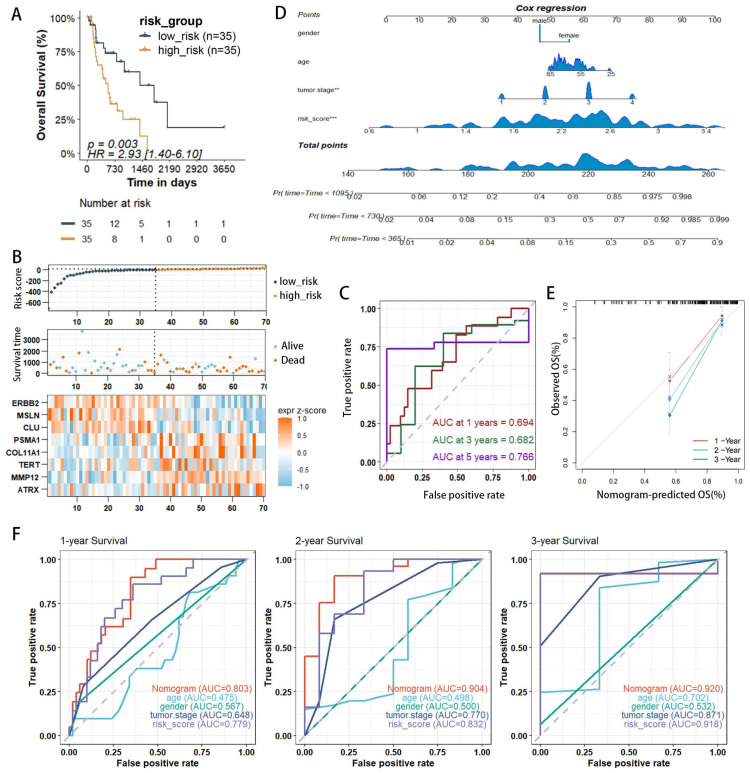
External validation of risk score prediction performance using the GEO cohort and a nomogram predicting overall survival (OS) in TCGA-EAC patients. (**A**) Kaplan-Meier (KM) survival curves for OS comparing high- and low-risk groups. (**B**) Distribution of risk status (high or low), survival outcomes in the GSE13898 cohort of the GEO database, and expression profiles of eight PANoptosis-associated risk genes. (**C**) Receiver operating characteristic (ROC) curves evaluating the predictive accuracy of the risk score within the GEO cohort. (**D**) Nomogram integrating clinical characteristics and PANscores derived from the TCGA dataset. (**E**) Calibration curves for the nomogram within the TCGA cohort. (**F**) Multi-indicator ROC curve analysis in the TCGA database.

The validation set utilized the GSE13898 dataset from the GEO database. Applying the risk model revealed survival curves that demonstrated differences in survival. Similar to the training set results, the survival status and risk plots (Figure 4A, B) also showed an inverse correlation between the risk score and patients' overall survival rates. ROC curves were used to evaluate the diagnostic efficacy of the prognostic model. The model yielded AUC values for 1-, 3-, and 5-year survival rates of 0.694, 0.682, and 0.766, respectively (Figure 4C). The findings from the validation set were consistent with those of the training set, underscoring the risk model's robust predictive power.

Given the capability of nomograms to quantitatively predict patient prognoses and guide clinical decision-making, nomograms incorporating statistically significant multivariate Cox regression factors, such as staging and risk scores, were constructed to predict the prognosis of EAC patients (Figure 4D). These nomograms achieved a C-index of 0.783 and demonstrated that the risk score had a more substantial impact on prognosis than other clinical characteristics, including age and staging. Calibration curves confirmed the nomograms' high reliability (Figure 4E). In the survival analysis, the ROC curves for the nomograms showed AUC values of 0.803, 0.905, and 0.920 for 1-, 2-, and 3-year survival rates, respectively (Figure 4F). Furthermore, when compared to AUC values for age, gender, and staging, the risk score model provided superior predictive accuracy, similar to results presented in Figure 3H. Additionally, with the highest AUC values across all categories, the nomogram demonstrated enhanced predictive performance over the risk score model, underscoring its superior capability in risk assessment.

### WGCNA identifies core gene clusters associated with risk factors

**Figure 5:**
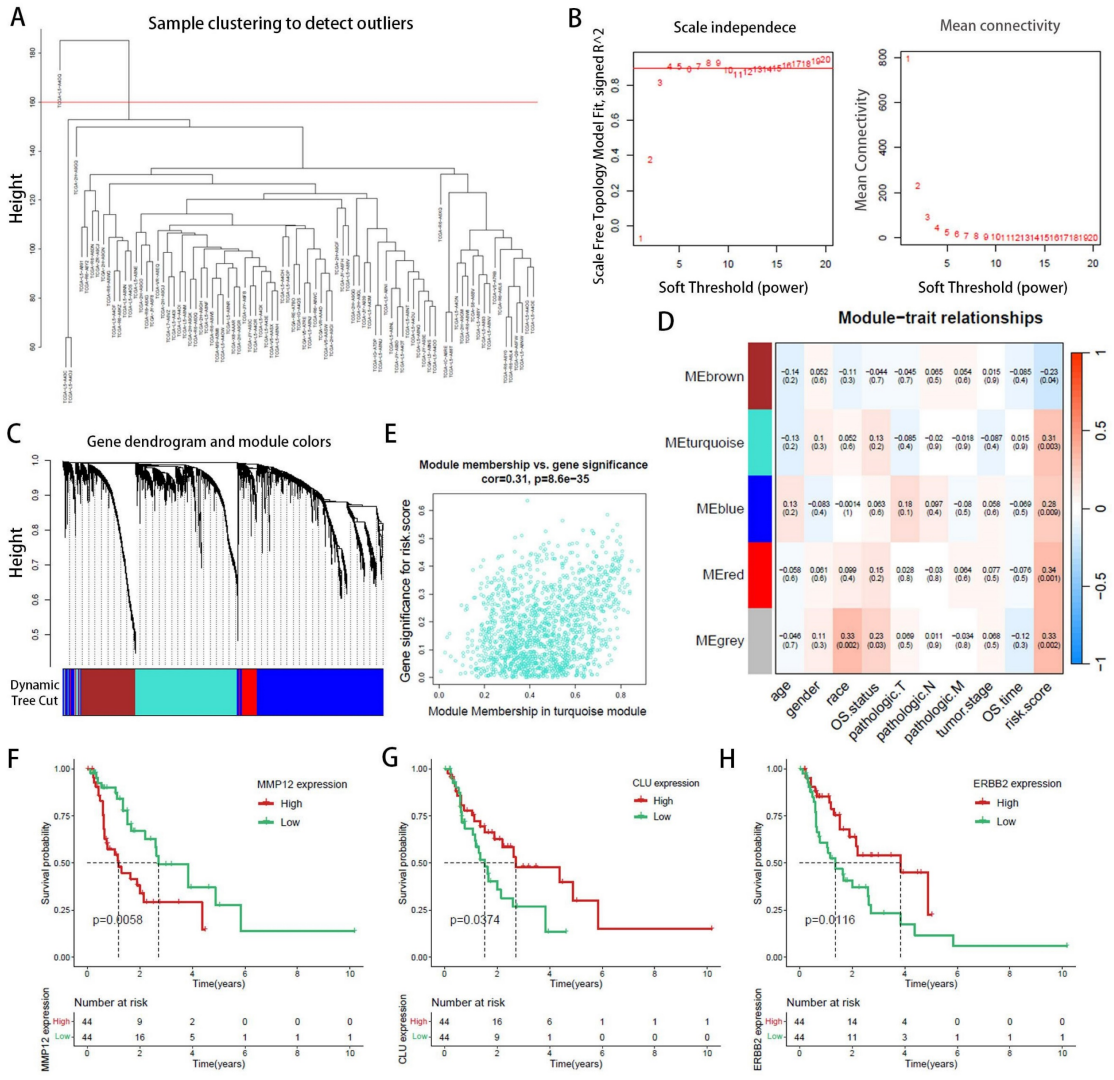
Weighted Gene Co-expression Network Analysis (WGCNA) identification of PANoptosis-related hub genes. (**A**) Dendrogram of samples. (**B**) Selection of an optimal power index for constructing a scale-free co-expression network. (**C**) Tree diagram branches representing different gene modules. (**D**) Heatmap displaying the correlation coefficients and associated p-values for each gene module with the risk score. (**E**) Scatterplot illustrating the correlation between the turquoise module and the PANoptosis gene module. (**F-H**) Kaplan-Meier survival curves for three hub genes.

**Figure 6:**
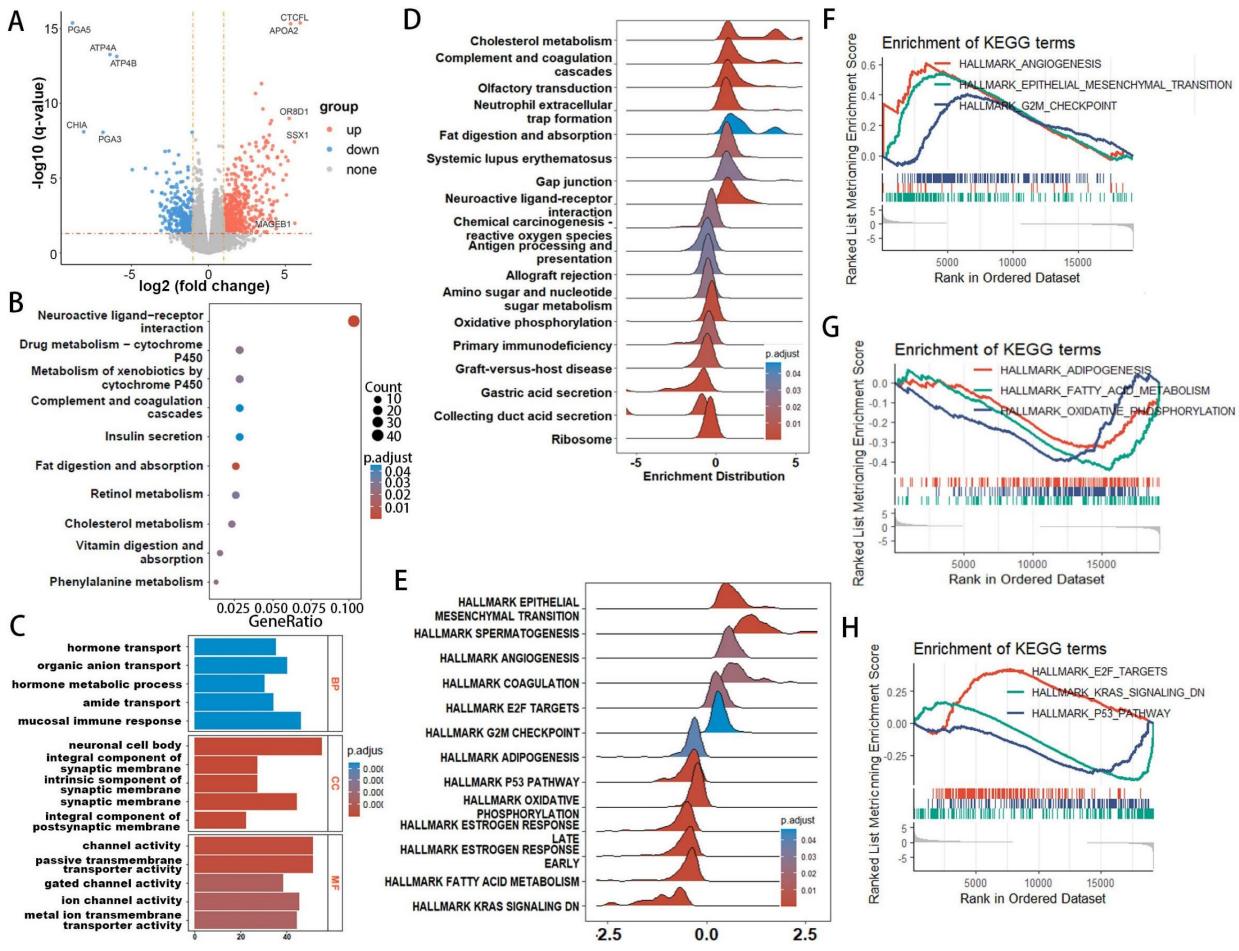
Functional enrichment analysis of genes associated with risk-score groups in EAC. (**A**) Volcano plot illustrating differentially expressed genes between high- and low-risk groups. (**B**) Kyoto Encyclopedia of Genes and Genomes (KEGG) pathway analysis. (**C**) Gene Ontology (GO) analysis for biological processes, molecular functions, and cellular components. (**D, E**) Ridge plot demonstrating the expression distribution across KEGG (**D**) and HALLMARK (**E**) pathways as analyzed by Gene Set Enrichment Analysis (GSEA). (**F-H**) Gene Set Enrichment Analysis (GSEA) reveals significant associations of the high-risk group with lipid metabolism-related processes.

WGCNA elucidates the transcriptomic organization and the relationships between gene clusters and external biological traits. Using the TCGA EAC dataset, WGCNA identified key PANoptosis gene modules associated with clinical characteristics (Figure 5A). An optimal soft-thresholding power of 8 was selected to construct a scale-free network (Figure 5B), and dynamic tree cutting facilitated the clustering of similar modules, identifying five distinct modules in total (Figure 5C). Excluding the grey module, which contains genes not assigned to any module and thus could not be effectively clustered, the red and turquoise modules demonstrated the highest correlation with the risk score (R2=0.34, p=0.001 and R2=0.31, p=0.003, respectively) (Figure 5D). The red module showed a correlation coefficient (cor) of 0.18, p=0.0049 (Supplementary Figure 1) and was excluded due to its low correlation, the turquoise module, with a cor of 0.31, p<0.001 (Figure 5E), indicated significant relevance. All 572 genes in the turquoise module were analyzed and intersected with 8,776 differentially expressed genes (DEgenes), 624 prognostic-related genes (PRGs), and 936 risk-related genes (Figure 6A,2C). This analysis led to the identification of three highly relevant prognostic-related DEPRGs associated with risk factors: MMP12, CLU, ERBB2. Survival analysis of RNA expression levels revealed that the expression of these three genes was significantly associated with prognosis: MMP12 was negatively correlated (Figure 5F), while CLU (Figure 5G) and ERBB2 (Figure 5H) exhibited positive correlations.

### GO, KEGG, and enrichment analysis

Given the thorough evaluation of the prognostic value of the prognostic-related DEPRGs-related risk model, we sought to explore underlying mechanisms. Differential expression analysis was conducted on the two risk groups identified in the previous section, revealing 614 upregulated and 322 downregulated genes in the high-risk group (Figure 6A). To probe the differences in biological functions and pathways between the high- and low-risk groups, further enrichment analysis was undertaken. KEGG analysis revealed that the DEGs were associated with pathways such as neuroactive ligand-receptor interaction, drug metabolism, and cholesterol metabolism (Figure 6B). GO enrichment analysis indicated that these DEGs were involved in hormone transport and metabolism, mucosal immune response, protein-lipid complex remodeling, and cholesterol absorption (Figure 6C). ORA enrichment analysis visualized both upregulated and downregulated pathways, showing significant upregulation in lipoprotein metabolism, metabolism of fat-soluble vitamins, and lipid regulation pathways, along with a trend of downregulation in gastric acid secretion and digestive functions across GO, KEGG, and REACTOME datasets (Supplementary Figure 2-4).

GSEA explored the enrichment of genes in KEGG pathway collections (Figure 6D), where pathways such as cholesterol metabolism and fat digestion and absorption were significantly upregulated in the high-risk group, while antigen processing and presentation, primary immunodeficiency, and metabolism of amino sugars and nucleotide sugars were significantly downregulated. HALLMARK pathway GSEA analysis (Figure 6E) showed significant upregulation of pathways related to epithelial-mesenchymal transition (EMT), angiogenesis, G2M checkpoint, and E2F targets in the high-risk group, while pathways related to oxidative phosphorylation, KRAS signaling, p53 pathway, and estrogen response were significantly downregulated (Figure 6F-H). GSVA enrichment analysis (Supplementary Figure 5-7) also demonstrated similar results.

### Immune infiltration analysis

**Figure 7:**
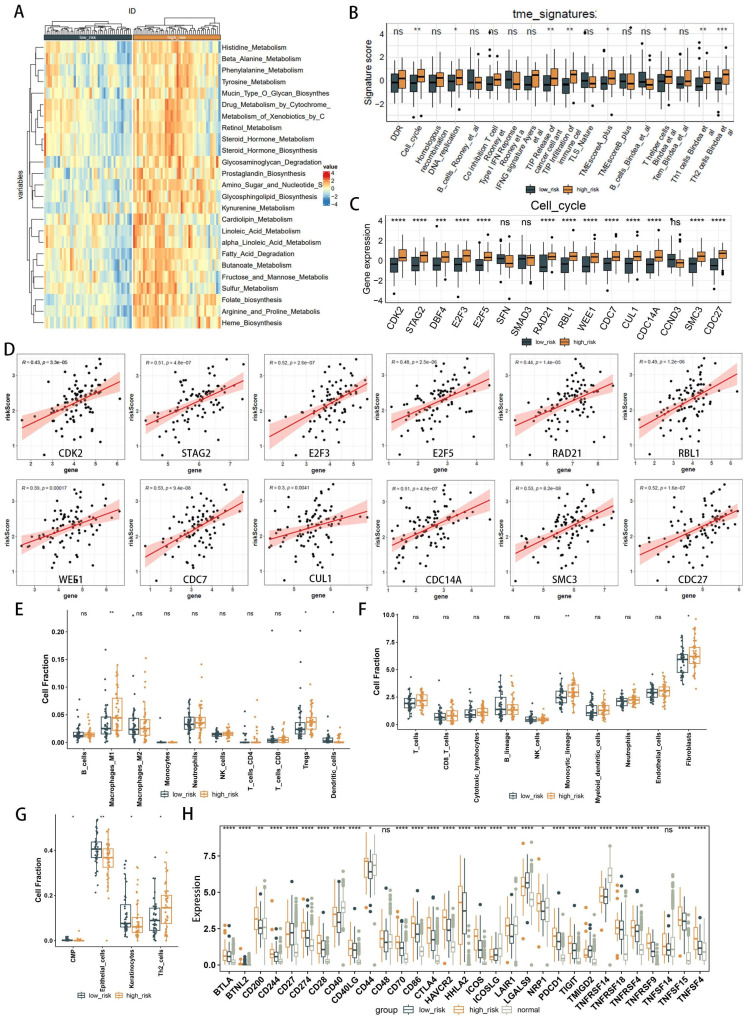
Comparative analysis of immune infiltration in high- and low-risk groups. (**A**) Heatmap illustrating metabolic and immune functional disparities. (**B**) Boxplot depicting differences in Tumor Microenvironment (TME) signatures. (**C**) Boxplot of differentially expressed genes related to the cell cycle. (**D**) Correlation plots illustrating the relationship between risk score and expression of cell cycle-related DEGs. (**E-G**) Analysis of TME cell infiltration abundances in high- and low-risk groups, evaluated using Quantiseq (**E**), MCPcounter (**F**), and xCell (**G**) methods within the TCGA cohorts. (**H**) Differential analysis of 22 types of immune cell infiltration, with significance levels denoted as *p < 0.05; **p < 0.01; ***p < 0.001; ****p < 0.0001.

The results of various enrichment analyses strongly suggest a close relationship between metabolic immune dysfunction and the high- and low-risk groups of EAC. Initially, we evaluated the immune characteristics related to the metabolic phenotype, finding significant differences in the metabolic immune spectra between high- and low-risk groups, consistent with previous results (Figure 7A, Supplementary Figure 8). Analysis of the TME immunophenotype revealed significant upregulation in the high-risk group in key processes of the tumor immunophenotype (TIP), including cell cycle and pathways related to Th1 and Th2 cells, tumor antigen release, and immune cell infiltration into tumors (Figure 7B). Further examination of gene expression in these pathways identified cell cycle-related genes as showing the most significant differential expression between high- and low-risk groups (Figure 7C, Supplementary Figure 9-12), with gene expression in the cell cycle gene set correlated with the risk score as shown in Figure 7D, including significant associations with high risk for genes like CDK2, STAG2, E2F3, E2F5, RAD21, RBL1, WEE1, CDC7, CUL1, CDC14A, SMC3, CDC27, and HELLS.

Immune cell composition was calculated using deconvolution methods. The Quantiseq algorithm revealed higher immune infiltration activity of macrophages and regulatory T cells (Tregs) in the high-risk group, with decreased infiltration activity of dendritic cells (Figure 7E). The MCPcounter algorithm identified higher immune infiltration activity of the monocyte lineage and fibroblasts in the high-risk group (Figure 7F). The xCell algorithm showed lower immune infiltration activity of epithelial cells in the high-risk group (Figure 7G). Considering immune checkpoints and ligands as potential therapeutic targets, we studied the expression of 31 immune checkpoint molecules across different groups (Figure 7H). Clinical immunotherapy markers, such as PD-L1 (CD274), CTLA4, and most immune checkpoint molecules, were found to be highly expressed in the high-risk group. These findings suggest that metabolic-related risk score grouping could serve as a potential biomarker for immune checkpoint blockade therapy.

### Core gene functional study and drug sensitivity prediction

**Figure 8:**
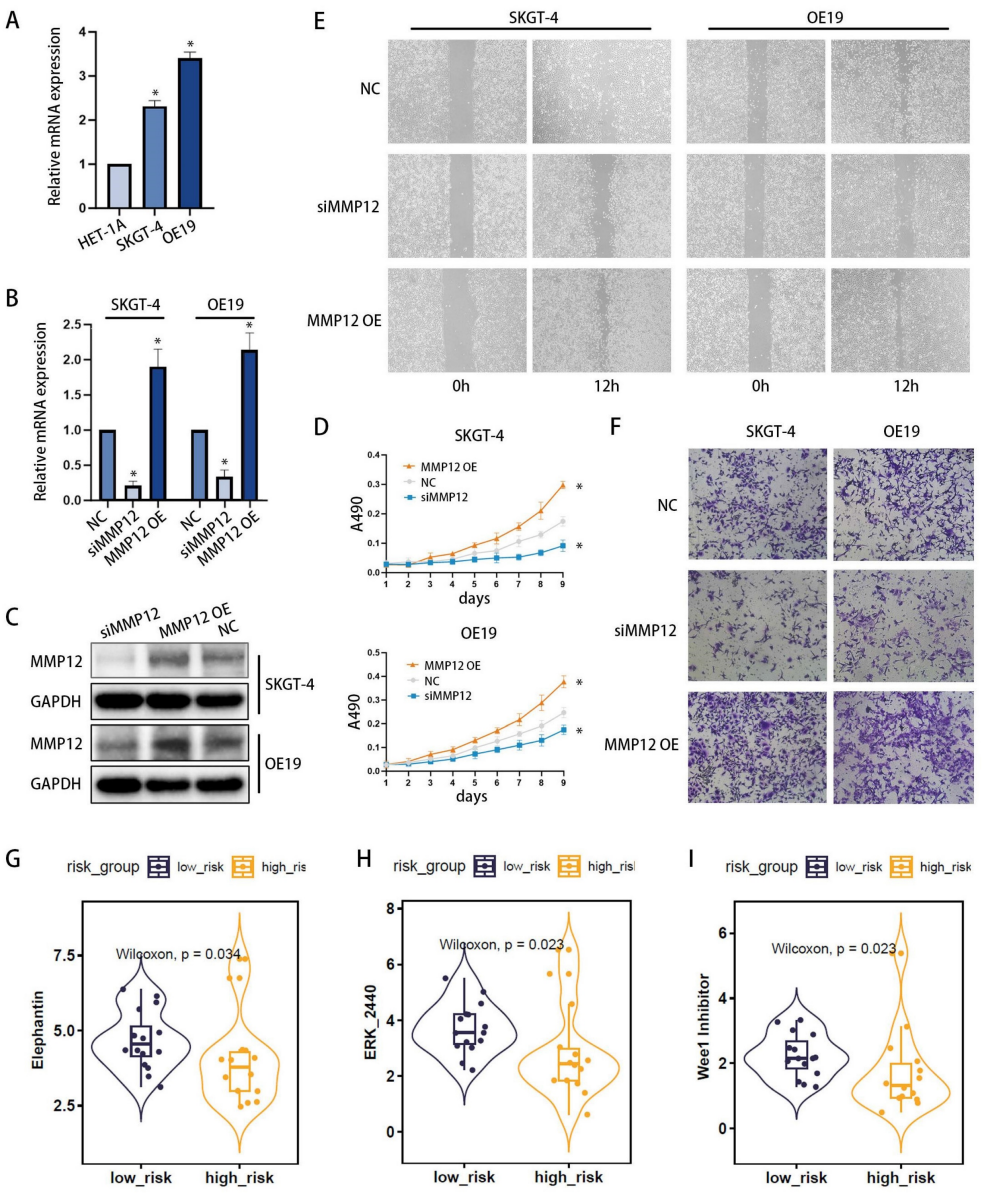
MMP12 Expression and Functional Analysis in EAC Cells, with Associated Drug Sensitivity Studies. (**A**) qPCR analysis of MMP12 expression in normal esophageal cells (HET-1A), well-differentiated EAC cell line (SKGT-4), and poorly differentiated EAC cell line (OE19). (**B, C**) Knockdown and overexpression of MMP12 confirmed by quantitative RT-PCR (b) and Western blotting (WB) (c) in SKGT-4 and OE19 cells, respectively (*p < 0.05; **p < 0.01; ***p < 0.001). (**D**) Cell proliferation of MMP12-knockdown EAC cells assessed by CCK-8 assays. (**E, F**) Motility of MMP12-knockdown and overexpressed EAC cells evaluated using wound healing assays (**E**) and Transwell invasion assays (**F**). (**G**) Box-violin plots illustrating lower IC50 values for three drugs in high-risk groups, suggesting increased drug sensitivity.

Among the three core genes mentioned previously (Figure 5F-H), only MMP12 was found to be overexpressed in the high-risk group, with clear clinical significance. Consequently, we proceeded to investigate the role of MMP12 in EAC. Initially, the expression of MMP12 was examined using qPCR (Figure 8A) across different esophageal cell lines: poorly differentiated EAC cell line OE19, highly differentiated EAC cell line SKGT-4 and the normal human esophageal epithelial cell line HET-1A. The results revealed that MMP12 expression was highest in the OE19 cell line, intermediate in the SKGT-4 cell line, and lowest in the HET-1A cell line, indicating a clear correlation between MMP12 expression and the malignant progression of esophageal cells. Furthermore, to modulate MMP12 expression, we constructed small interfering RNA (siRNA) plasmids (siMMP12) to knock down MMP12, and overexpression plasmids (MMP12 OE) to upregulate its expression. Following transfection into OE19 and SKGT-4 cell lines, MMP12 expression levels were assessed using qPCR (Figure 8B) and Western blotting (WB) (Figure 8C), validating the effectiveness of the plasmids. Proliferation assays demonstrated that siMMP12 significantly reduced cell proliferation in both cell lines, while overexpressing MMP12 significantly enhanced their proliferation (Figure 8D). Moreover, scratch assays (Figure 8E) and Transwell invasion assays (Figure 8F) indicated that MMP12 downregulation substantially decreased the migration and invasion capabilities of the cells, whereas upregulation of MMP12 significantly increased these abilities.

Drug sensitivity analysis was conducted using the oncoPredict R package, identifying three drugs (Elephantin, ERK_2440, Wee1 Inhibitor) that exhibited significantly lower IC50 values in the high-risk group. This indicates enhanced efficacy within this group (Figures 8G-I).

## Discussion

Adenocarcinoma represents the most common histological subtype of Esophageal cancer in many Western countries with incidence rate rising rapidly^2^. Despite extensive investigations into distinct genetic drivers and prognostic factors, EAC patients still suffer from poor survival on account of undetected pathogenesis^4^. Clinicians are urgently seeking novel approaches to accurately predict prognosis and refine treatment decisions for patients with esophageal adenocarcinoma.

Cell death usually does not occur independently but in a mixed form because cells can undergo extensive crosstalk under pathological conditions^6^. Previous research indicates the existence of a composite cell death form involving pyroptosis, apoptosis, and necroptosis, termed PANoptosis^37^. PANoptosis as a component of the host's innate immune response, has been identified as a novel mechanism governing inflammatory programmed cell death^38^. Previous studies have demonstrated the dual role of PANoptosis in tumorigenesis and anti-tumor therapies^12^, ^14^, ^39^. However, the relationship between PANoptosis and EAC, especially the role of PRGs in tumor microenvironment and prognosis, remains unclear.

In our study, we comprehensively evaluated the PANoptosis-related gene profiles in EAC and identified 18 genes significantly associated with prognosis (prognostic-related DEPRGs). LASSO analyses screened genes to construct an 8-gene prognostic signature. Finally, the risk score was calculated based on the expression levels of ATRX, TERT, PSMA1, ERBB2, CLU, MMP12, MSLN, and COL11A1. Previous studies have established the association between some of these genes and EAC. The ERBB2 gene encodes a protein that belongs to the epidermal growth factor receptor family. Overexpression or amplification of ERBB2 has been linked to a worse prognosis and disease progression in various cancers, including esophageal adenocarcinoma^40^. The Telomerase Reverse Transcriptase (TERT) gene plays a crucial role in maintaining telomere stability and is essential for cellular immortalization. Promoter mutations of the TERT gene frequently occur in different cancers, including esophageal adenocarcinoma, and are associated with tumor aggressiveness and poor prognosis^41^. Matrix Metallopeptidase 12 (MMP12) encodes a metalloproteinase involved in extracellular matrix remodeling within the tumor microenvironment, contributing to cancer invasion and metastasis. Alterations in expression patterns of MMPS family members have been reported in esophageal adenocarcinoma^42^. COL11A1 (Collagen Type XI Alpha 1) encodes a collagen protein that participates in extracellular matrix composition. Abnormal collagen expression is associated with tumor aggressiveness across various cancers, including esophageal cancer^43^. These findings suggest that these genes hold potential as diagnostic and therapeutic targets for EAC.

According to the median risk score, we divided the patients into high-risk and low-risk groups. KM curves demonstrated significantly better outcomes for patients in the low-risk group compared to those in the high-risk group. ROC analyses confirmed the predictive efficacy of the risk score. Additionally, the risk score was validated as an independent prognostic factor through multivariate Cox regression analyses, with Hazard Ratio (HR) values exceeding 1, underscoring the model's reliability. Critically, the prognostic capabilities of the 8-gene signature were corroborated using an independent GEO EAC dataset (GSE13898). This means the 8-gene signature showed good performance for predicting EAC prognosis in both the internal and external validation cohorts. The distinctive feature of the risk score is its ability to individually assess and score each patient's condition. Among these 8 genes, ERBB2, CLU and MSLN were highly expressed in the low-risk group, while ATRX, TERT, PSMA1, MMP12, and COL11A1 were highly expressed in the high-risk group. So high-risk groups were characterized by elevated expression levels of most PRGs and poorer prognoses, therefore these patients may benefit from aggressive therapies, whereas more frequent monitoring and surveillance can aid in early disease detection. Nomograms are widely used as prediction tools in oncology, particularly for survival prediction^44^, ^45^. In our study, a nomogram model was established according to the risk score and other clinical characteristics. Calibration plots confirmed that actual survival rates closely matched those predicted by the nomogram, indicating its high predictive accuracy.

Given the thorough evaluation of the prognostic value of the prognostic-related DEPRGs-related risk model, we sought to explore underlying mechanisms. Through enrichment analysis, we found that lipid metabolism, mainly cholesterol metabolism, as well as fat digestion and absorption pathways were significantly enriched in the high-risk group, suggesting that lipid metabolism may be the main energy metabolism mode of cells with high PRGs expression. Previous studies have shown that lipid metabolism not only supports the metabolic needs of tumor cells but also affects the function of immune cells in the tumor microenvironment^46^. Several studies have found that heightened lipid metabolism in the tumor microenvironment can produce lipid immunosuppression metabolites that inhibit anti-tumor immunity of immune cells^47^. For instance, tumor cells are able to produce specific lipid metabolites through lipid metabolism, such as oxidized fatty acids and prostaglandin E2 (PGE2), which can directly inhibit the function of immune cells, such as reducing the activity of T cells, promoting the proliferation of regulatory T cells (Tregs), or promoting the polarization of macrophages to an immunosuppressive phenotype^46^. Our immune infiltration analysis also found that elevated expression of M1 macrophages and regulatory Tregs in the high-risk group, which are known for their immunosuppressive activity. M1 macrophages inhibit the release of the immune-stimulating factor IL-12 and diminish the tumor-killing effects of NK cells and cytotoxic CD4+ T cells^48^. Tregs suppress the excessive immune response by expressing inhibitory factors (CTLA4, secreting IL-10 and TGF-β) in the process of tumor immunity, and may also promote tumor cell immune escape^49^. These findings suggest that EAC cells with high PRGs expression could make full use of lipids in TME, reprograming the immunosuppressive microenvironment with immunosuppressive cells, which can assist tumor cell immune escape and subsequently result in a poorer survival rate. What's more, clinical immunotherapy markers, such as PD-L1 (CD274), CTLA4, and most immune checkpoint molecules, were found to be highly expressed in the high-risk group, which means that the high-risk group is more sensitive to immune checkpoint inhibitor therapy.

In the analysis of differentially expressed genes (DEGs) between high-risk and low-risk groups, when intersected with the first three gene sets (DEGs, PRGs, and WGCNA key genes), only MMP12 was consistently identified as a core gene, and whose prognostic trend aligns with its expression pattern. This study thus focuses on MMP12 due to its pivotal role in risk assessment. MMP12, primarily expressed by macrophages, degrades extracellular matrix (ECM) components, thereby facilitating the migration and invasion of cancer cells^50^. This degradation also remodels the tumor microenvironment, creating conditions that are more conducive to tumor progression. Additionally, MMP12 modulates the activity of growth factors and cytokines, such as VEGF and TGF-β, which play crucial roles in angiogenesis and inflammation within the tumor microenvironment^51^. MMP12's impact on the immune response within tumors can also contribute to an immunosuppressive environment that favors tumor growth^52^. The role of MMP12 in PANoptosis and its implications for EAC have gained increasing recognition. In the context of PANoptosis, MMP12 may regulate inflammation by degrading extracellular matrix components, which in turn affects the accessibility and activity of cell surface receptors, as well as modulates the maturation and secretion of pro-inflammatory cytokines, such as interleukin-1β (IL-1β) and IL-18^53^. Additionally, MMP12 influences cellular membrane stability, leading to increased permeability and facilitating PANoptosis^54^. In EAC, MMP12 plays a multifaceted role, not only promoting tumor invasion and metastasis by degrading extracellular matrix components but also contributing to the creation of a microenvironment that favors tumor cell survival and progression^55^. In our study, GSEA analysis suggested that the epithelial-mesenchymal transition (EMT) and angiogenesis-related pathways were enriched in the high-risk group, so we reasonably inferred that MMP12 may promote the invasion and metastasis of EAC cells through mediating EMT and promoting angiogenesis. Subsequently, we initially validated this perspective through in vitro experiments. The q-PCR and Western blotting confirmed that the lower the degree of cell differentiation, the higher the expression level of MMP12 in EAC cells. Additionally, through cell proliferation assays, scratch tests and Transwell invasion experiments, we further observed the role of MMP12 in enhancing the proliferation, migration and invasion capabilities in EAC cells. Therefore, MMP12 may be an important therapeutic target for EAC. Future research will continue to delve into the detailed mechanisms by which MMP12 plays its role.

Finally, drug sensitivity analysis identified three drugs (Elephantin, ERK_2440, and Wee1 Inhibitor) as being more sensitive in the high-risk group, suggesting that our risk model has the potential to predict the effectiveness of drug therapy. By predicting the best individualized treatment based on the patient's risk gene expression, we can improve treatment outcomes.

Although this study has made some progress in the field of EAC, we recognize several limitations. Firstly, while ERBB2 and CLU expression were found to be elevated in tumor tissues compared to non-tumor tissues, their expressions were lower in the high-risk group compared to the low-risk group, indicating an inconsistency between prognostic trend and expression pattern. These inconsistencies may arise from both technical and biological factors. Technically, variations in sample handling, the choice of technology platforms (e.g., microarrays vs. RNA sequencing), data normalization, and statistical analysis can all impact the accuracy of gene expression measurements. Biologically, factors such as tumor heterogeneity, individual patient characteristics (including genetic background, age, and sex), environmental influences (such as lifestyle and diet), disease stage, and comorbidities can all modulate gene expression and influence its correlation with prognosis. Therefore, the risk model we constructed requires further validation and refinement in larger-scale, prospective, and multi-center studies in the future. Additionally, although we observed significant differences in the expression of cell cycle-related genes between the high-risk and low-risk groups, suggesting their important roles in the TME and metabolic immune processes, the specific mechanisms remain unclear. Lastly, while we have confirmed the key role of the core gene MMP12 in the occurrence and development of EAC, the interaction mechanisms between MMP12 and PANoptosis, as well as its detailed involvement in metabolic immunity, are issues that we need to address in future research.

## Conclusion

In conclusion, our study builds models based on public databases such as TCGA, GTEx and GEO, which contain a large number of samples and relatively complete clinical information, providing a reliable data foundation. The model we employed focuses on specific genes, and the designed scoring system is not only easy to operate and cost-effective but also contributes to the advancement of precision medicine and clinical decision-making. The outcomes of this study further emphasize the potential of PRGs in regulating cellular metabolic immunity in the EAC tumor microenvironment, paving new pathways for future EAC treatment research.

## Supplementary Material

Supplementary figures and table 2.

Supplementary table 1.

## Figures and Tables

**Figure 1:**
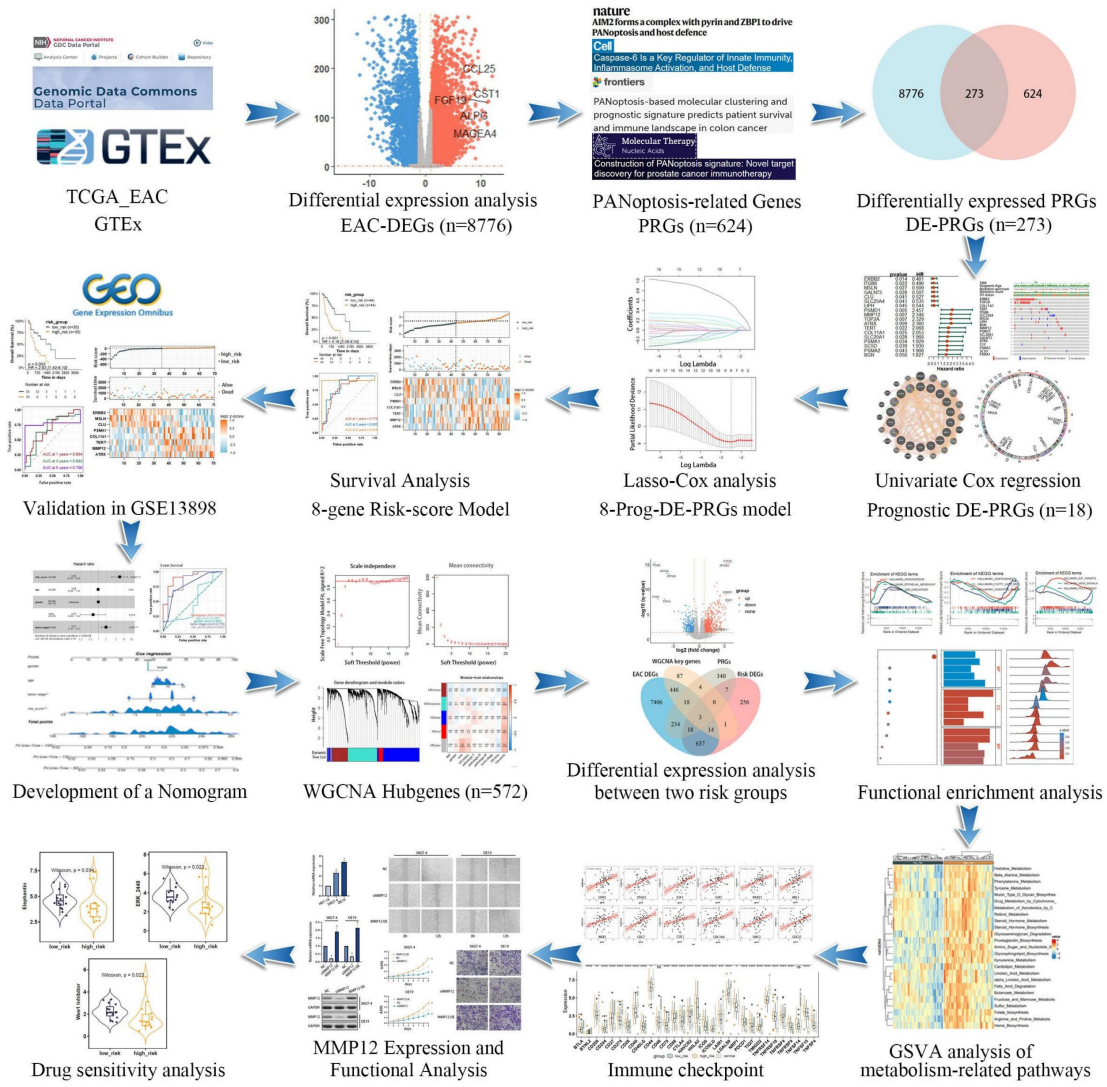
Study workflow diagram.
