# Supplementary material for: Deciphering the Prognostic Landscape of Esophageal Adenocarcinoma: A PANoptosis-Related Gene Signature
Source: J Cancer. 2025 Jan 1;16(1):183–200. doi: 10.7150/jca.102180 (PMC11660136; doi:10.7150/jca.102180)
Supplement: Supplementary file 1 — Supplementary figures and table 2. [file jcav16p0183s1.pdf]

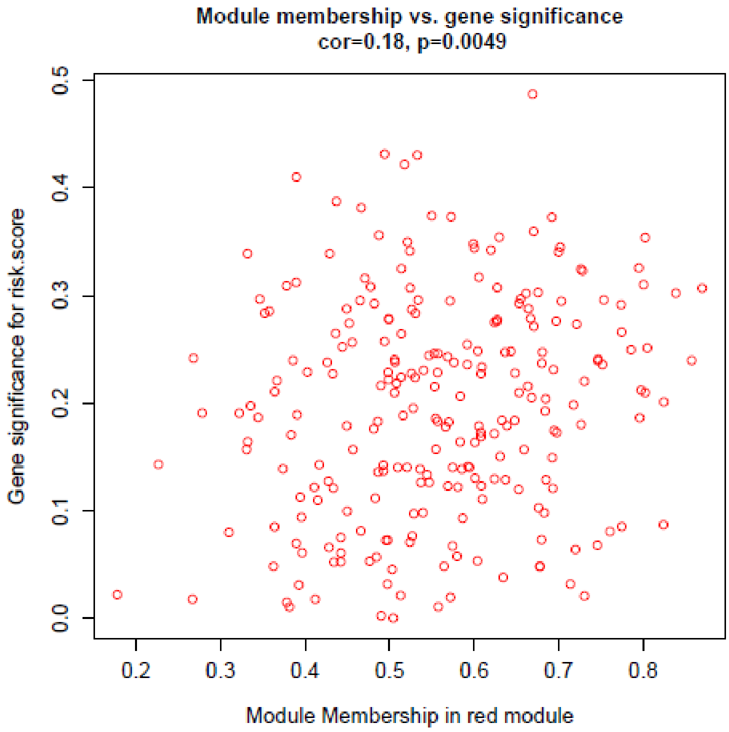

Supplementary Figure 1: Scatterplot illustrating the correlation between the red module and the PANoptosis gene module (cor=0.18, P=0.0049).

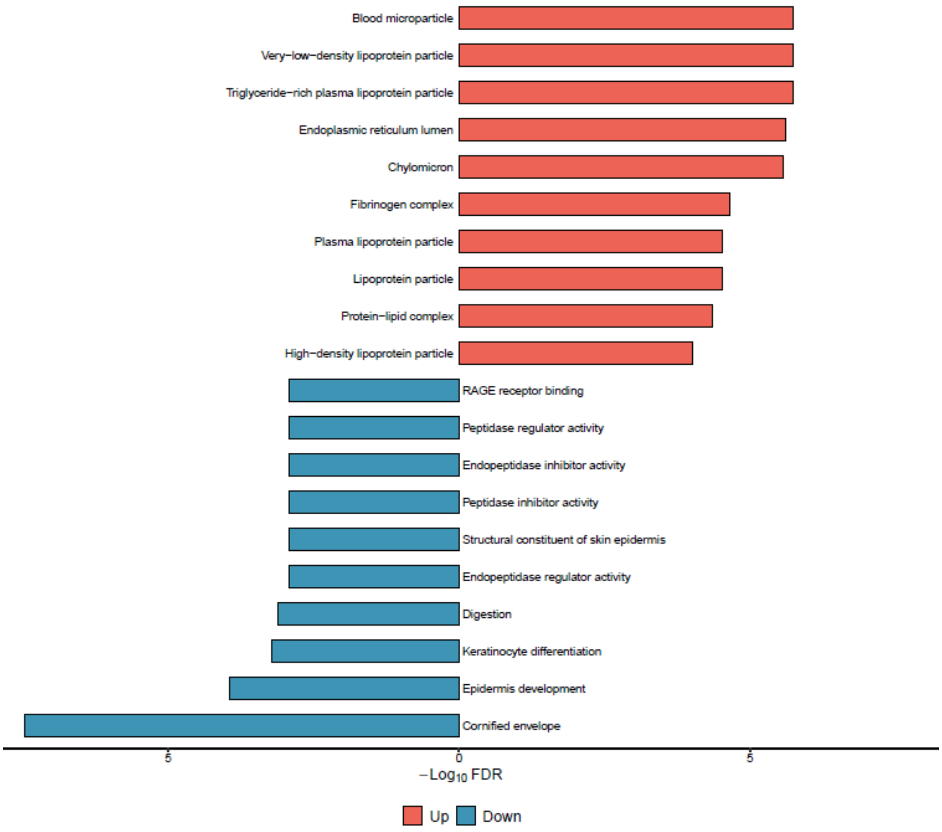

Supplementary Figure 2: Overrepresentation Analysis (ORA) enrichment of Gene Ontology (GO) terms.

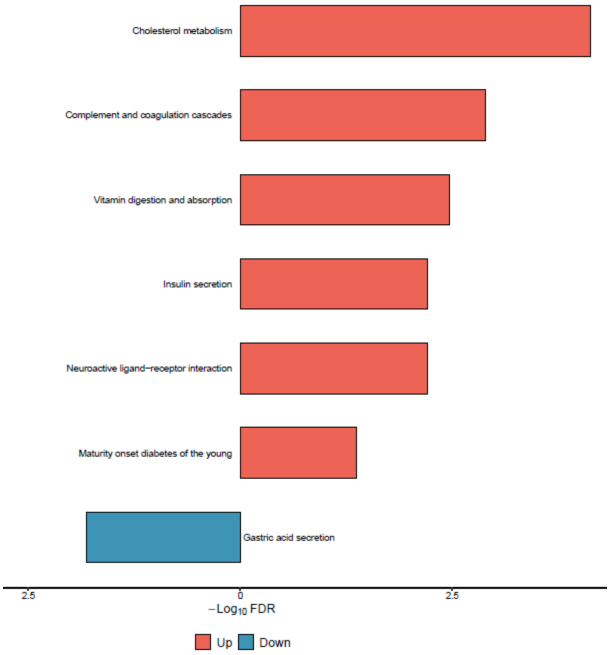

Supplementary Figure 3: Overrepresentation Analysis (ORA) enrichment of Kyoto Encyclopedia of Genes and Genomes (KEGG) pathways.

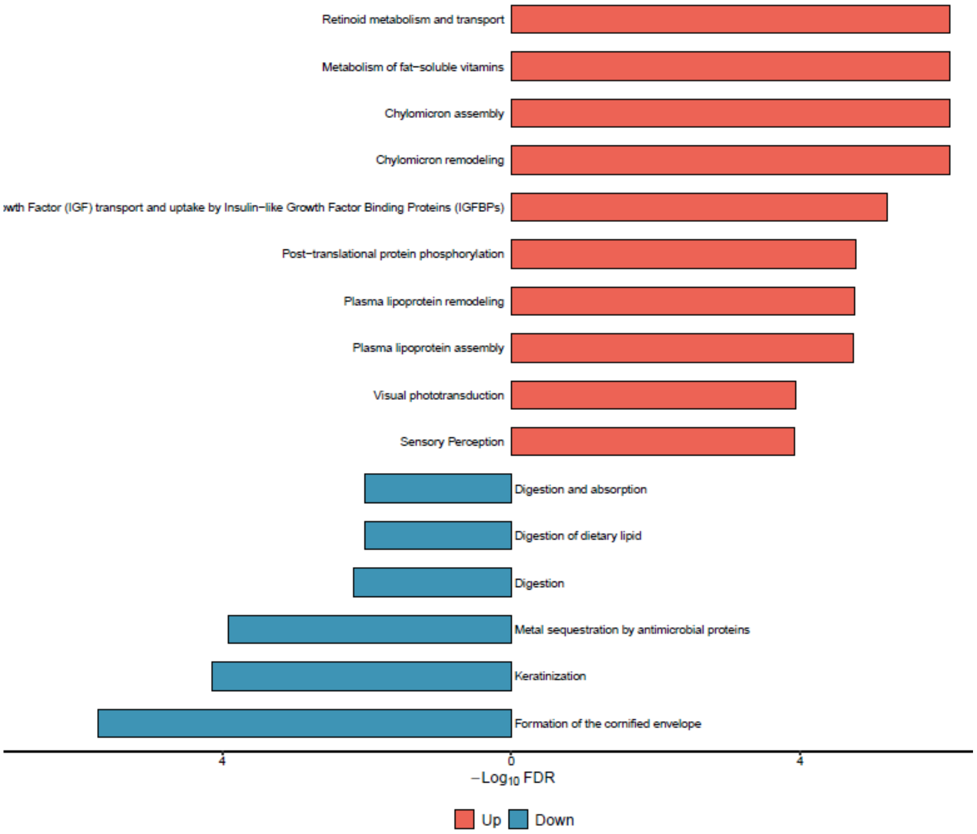

Supplementary Figure 4: Overrepresentation Analysis (ORA) enrichment of REACTOME pathways.

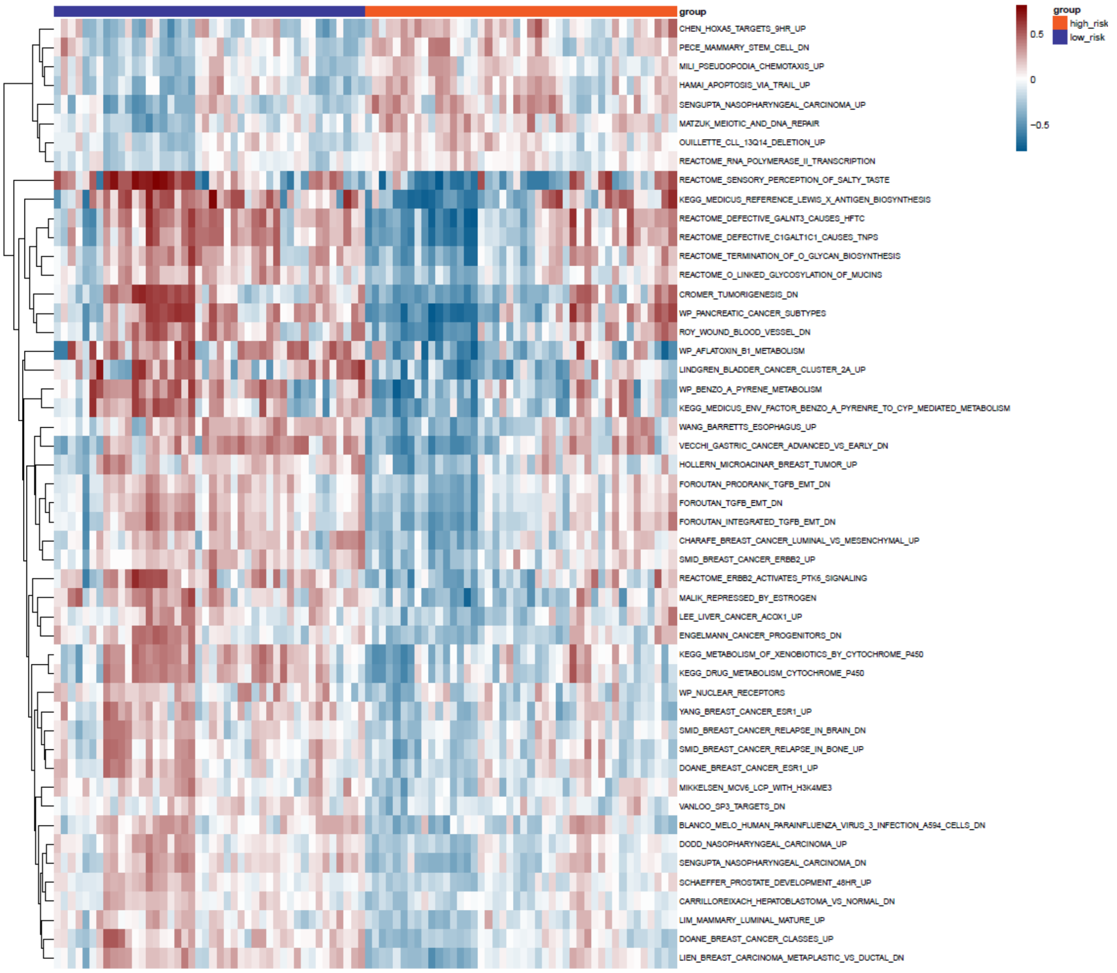

Supplementary Figure 5: Gene Set Variation Analysis (GSVA) heatmap for C2 gene sets.

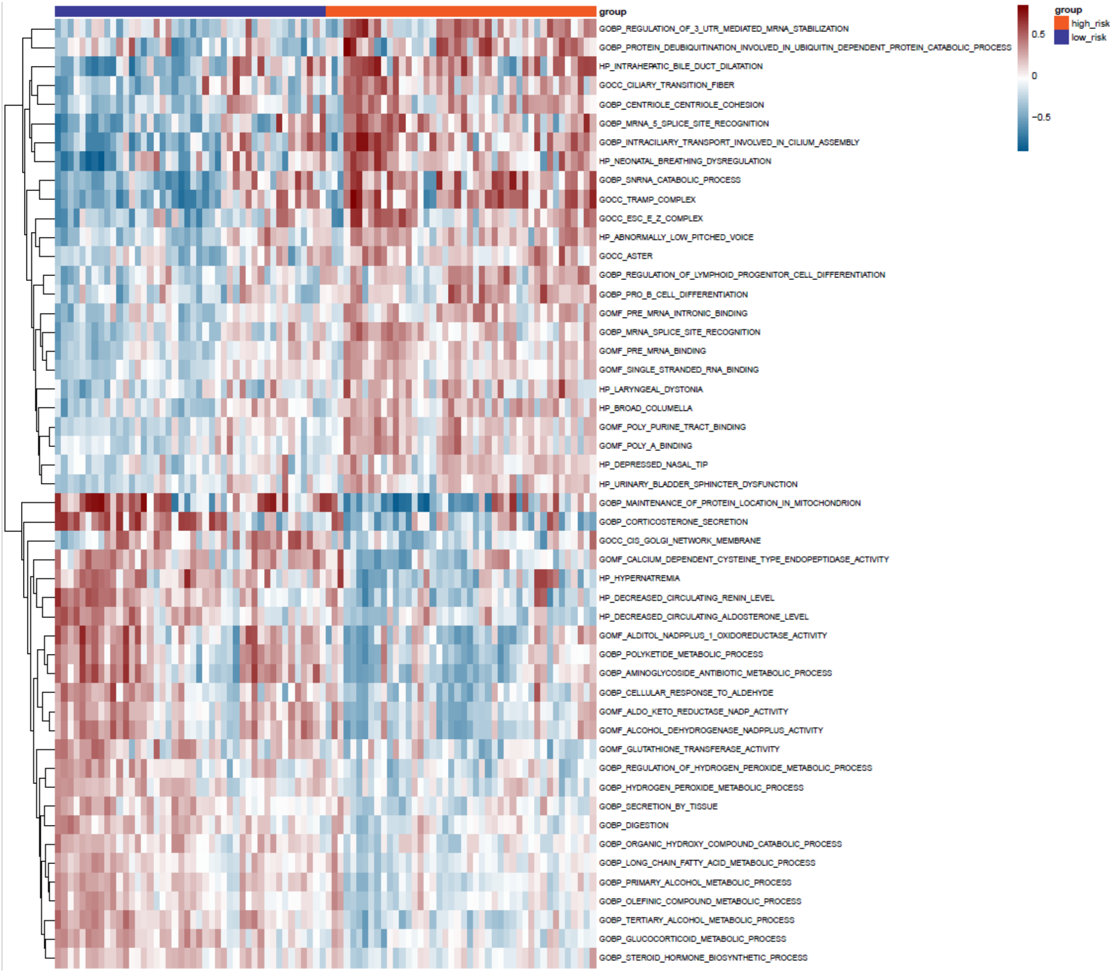

Supplementary Figure 6: Gene Set Variation Analysis (GSVA) heatmap for C5 gene sets.

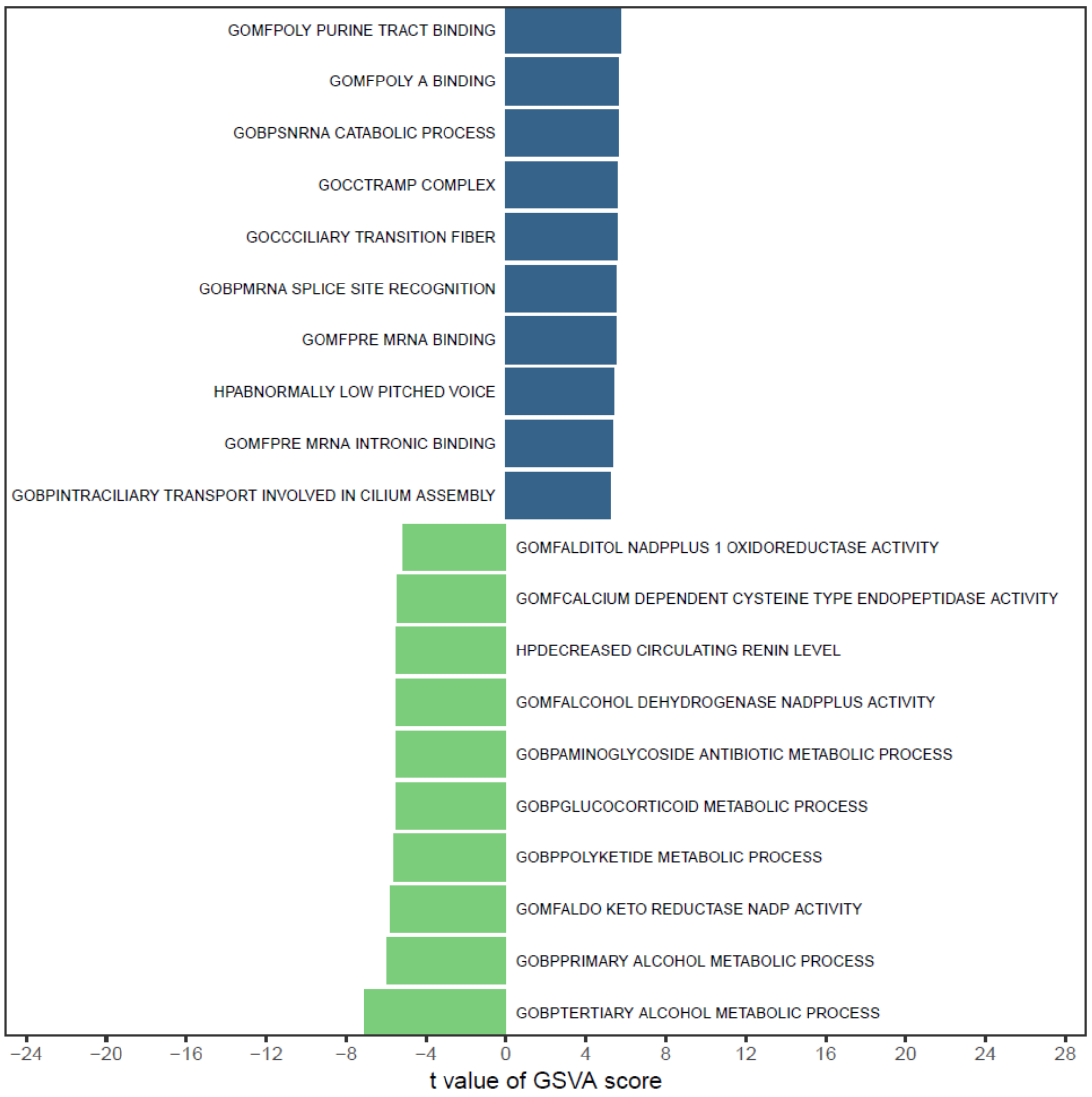

Supplementary Figure 7: Bar plot comparing the top 20 enriched C5 gene sets using GSVA.

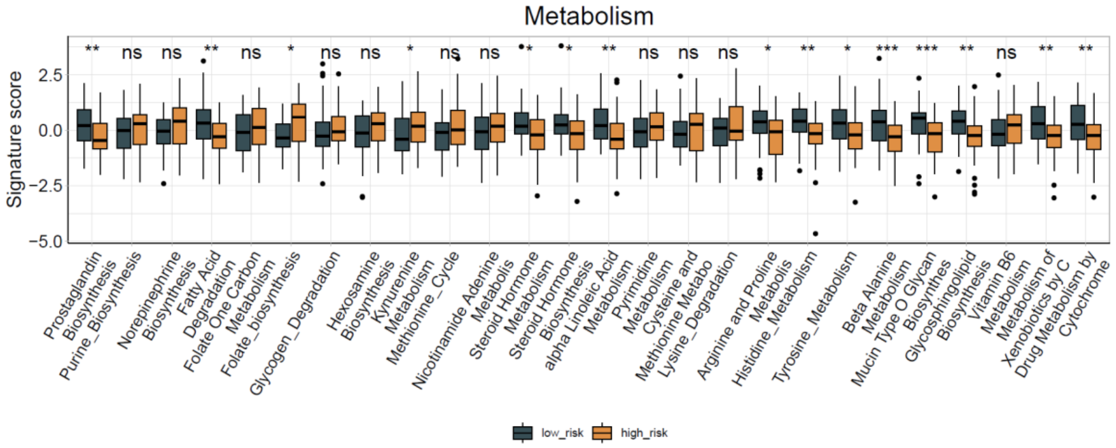

Supplementary Figure 8: Significant differences in the metabolic-immune spectra between high- and low-risk groups.

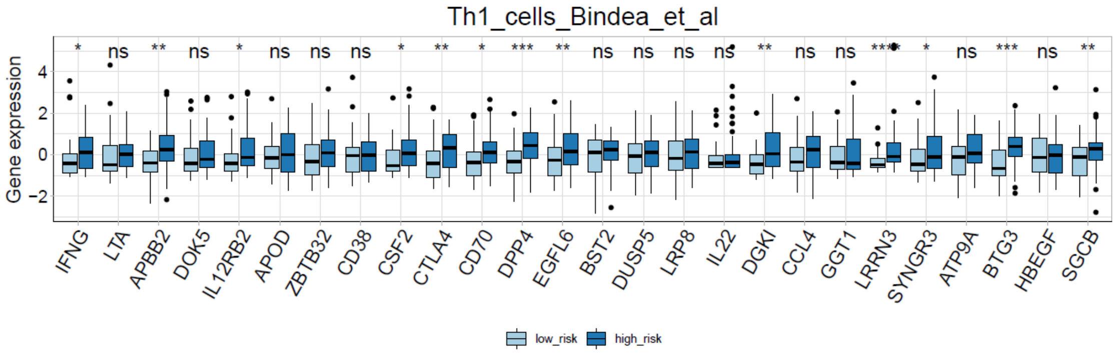

Supplementary Figure 9: Differential expression of Th1 cell-related genes between high- and low-risk groups.

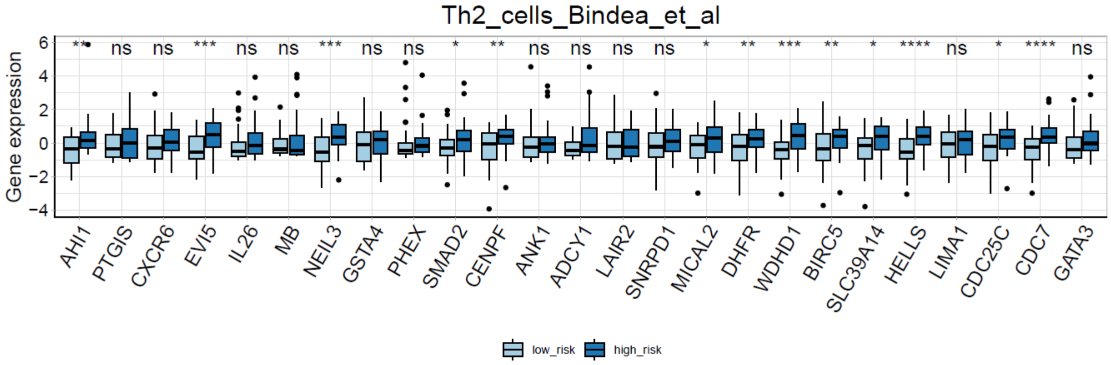

Supplementary Figure 10: Differential expression of Th2 cell-related genes between high- and low-risk groups.

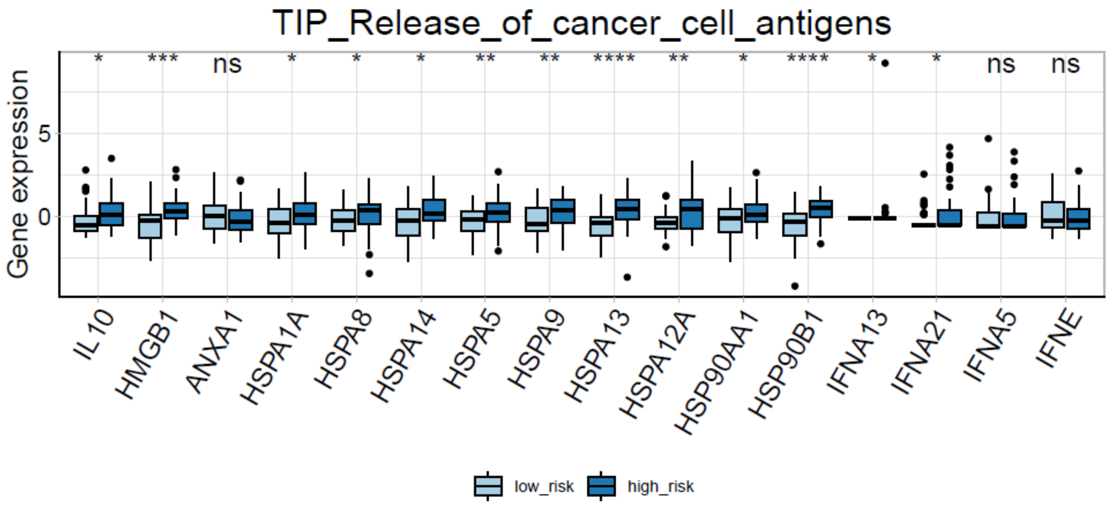

Supplementary Figure 11: Differential expression of genes related to the TIP (Tumor Immune Phenotype) mechanism of cancer cell antigen release between high- and low-risk groups.

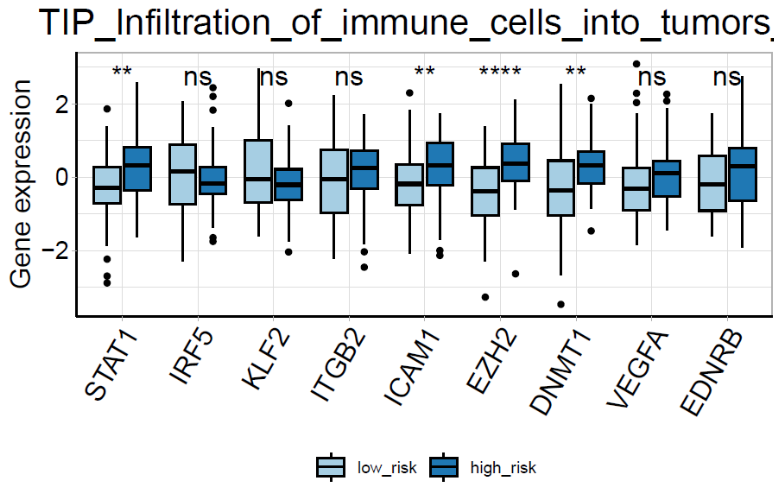

Supplementary Figure 12: Differential expression of genes related to the TIP mechanism of immune cell infiltration into tumors between high- and low-risk groups.

Supplementary Table2: The 8 PRGs with prognostic relevance (ATRX, TERT, PSMA1, ERBB2, CLU, MMP12, MSLN, and COL11A1) that were used to construct a risk score model.

**gene**	**coef**
ATRX	0.558587
TERT	0.210979
PSMA1	0.115473
ERBB2	-0.09513
CLU	-0.09145
MMP12	0.07644
MSLN	-0.04294
COL11A1	0.006516
